# Plan Competitions and the Difficulties of Architects: An Example

**Published:** 1904-06-25

**Authors:** 


### PLAN COMPETITIONS AND THE DIFFICULTIES OF ARCHITECTS: AN EXAMPLE.

The Town Council of Perth advertised for, or invited, a competition for plans of an isolation hospital to cost £11,000, and six architects sent in designs. Mr. Aldwinkle was appointed assessor, and he placed the plans in the following order:—1st Mr. Young, of Perth; 2nd Mr. Heiton, of Perth, and 3rd Mr. Thomson, of Brighton. Unfortunately, however, Mr. Aldwinkle is of opinion that the first premiumated design cannot be built for less than £12,500, and the question naturally arises whether the premium should be given to Mr. Young. One of the councilors, Mr. Alexander, said his opinion was that it was unfair that the prize should be given to a plan which would not work out at the sum named in the conditions, and manifestly this opinion is correct, because it is quite possible, even probable, that some of the competitors tried all they knew to keep the cost down, and they might in doing so have lost all chance of a premium. These questions often crop up at competitions, and to us it seems that it is no more right to give the premium to an architect who fails in this condition than to one who fails in some other condition; but then it is impossible to say what the building will cost until the contractors' tenders are in. A committee should never land itself in this position. If the conditions have been clearly drawn out and including everything necessray, and excluding everything unnecessary, and the estimated expense based on their expressed requirements, there should not be much difficulty in keeping within the estimate. But were the conditions carefully drawn out in this case, and were the instructions to the assessor explicit? These are questions we have no means of answering.

